# Association of Low Serum l-Carnitine Levels with Peripheral Arterial Stiffness in Patients Who Undergo Kidney Transplantation

**DOI:** 10.3390/nu11092000

**Published:** 2019-08-24

**Authors:** Yu-Hsien Lai, Ming-Che Lee, Guan-Jin Ho, Chin-Hung Liu, Bang-Gee Hsu

**Affiliations:** 1 Division of Nephrology, Hualien Tzu Chi Hospital, Buddhist Tzu Chi Medical Foundation, Hualien 97004, Taiwan; 2 Program in Pharmacology and Toxicology, Tzu Chi University, Hualien 97004, Taiwan; 3 Department of Surgery, Hualien Tzu Chi Hospital, Buddhist Tzu Chi Medical Foundation, Hualien 97004, Taiwan; 4 School of Medicine, Tzu Chi University, Hualien 97004, Taiwan; 5 Department of Pharmacology, Tzu Chi University, Hualien 97004, Taiwan

**Keywords:** l-carnitine, peripheral arterial stiffness, kidney transplantation, brachial–ankle pulse wave velocity

## Abstract

l-carnitine is an important co-factor in fatty-acid metabolism, and its deficiency is associated with insulin resistance, which is independently associated with arterial stiffness. This study evaluated the relationship between serum l-carnitine level and peripheral arterial stiffness (PAS) in kidney transplantation (KT). Fasting blood samples were collected from 65 patients who underwent KT. We measured the brachial–ankle pulse wave velocity, and 36 patients (55.4%) had PAS. Patients with PAS had a significantly higher percentage of diabetes (*p* = 0.001), hypertension (*p* = 0.033), and metabolic syndrome (*p* = 0.044); higher waist circumference (*p* = 0.010), systolic blood pressure (*p* = 0.002), serum triglyceride level (*p* = 0.040), insulin level (*p* = 0.002), and homeostasis model assessment of insulin resistance (*p* = 0.002); lower high-density lipoprotein cholesterol (*p* = 0.036) and serum l-carnitine levels (*p* < 0.001); older age (*p* = 0.041); and a longer KT duration (*p* = 0.025) than those without PAS. Statistical analysis revealed an independent association between PAS in KT and KT duration (95% confidence interval (CI): 1.003–1.054, *p* = 0.029) and serum l-carnitine levels (95% CI: 0.842–0.998, *p* = 0.044). The area under the receiver operating characteristic curve indicated that the diagnostic power of l-carnitine to predict PAS was 0.789 (95% CI: 0.670–0.881, *p* < 0.001). Serum-free l-carnitine level is negatively associated with PAS in patients who undergo KT.

## 1. Introduction

Cardiovascular disease (CVD) is the primary cause of mortality among patients with end-stage renal disease and those who undergo kidney transplantation (KT) [1]. Atherosclerosis plays a significant role in cardiovascular events and all-cause mortality [2]. Although atherosclerosis is improved and prevented after KT, CVD prevalence is still higher in patients who undergo KT than in the general population [3]. Therefore, it is important to reduce CVD risk factors after KT.

Arterial stiffness is one of the major modifiable CVD risk factors that are independently related to CVD occurrence in patients who undergo KT [3,4] Arterial stiffness is reportedly related to insulin resistance and metabolic syndrome [5], and it can be assessed by pulse wave velocity (PWV). PWV is a reliable and non-invasive method which represents predictive and categorized value for vascular dysfunction assessment [6,7,8]. Both brachial–ankle PWV (baPWV) and carotid–femoral PWV (cfPWV) can be used to determine arterial stiffness, which was proven to be associated with CVD as well [9,10,11]. In addition, baPWV can be measured easily and is a prognostic marker for peripheral arterial stiffness (PAS) [6,7].

l-carnitine, an essential enzyme co-factor in energy metabolism, is considered to be one of the target molecules of mitochondria [12]. It is also reportedly associated with insulin resistance [12,13]. The baPWV value was positively associated with changes in plasma l-octanoylcarnitine level in overweight middle-aged individuals after a three-year follow-up [14]. No previous studies investigated the association of serum-free l-carnitine and PAS by measuring baPWV value in patients who underwent KT. Cardiovascular events are some of the major causes of morbidity and mortality among patients who undergo KT; therefore, the present study aimed to determine the relationship between serum l-carnitine and PAS in patients who undergo KT.

## 2. Materials and Methods

### 2.1. Patients

Patients who underwent KT were recruited in a medical center in eastern Taiwan between April and June of 2013. All patients with an acute infection, pulmonary edema, heart failure, acute myocardial infarction, acute rejection, or malignancy at the time of blood sampling, or having an arterial–venous shunt (fistula or graft) in either limb were excluded from the study. The subjects were also excluded if they had a history of medication usage related to calcium, active vitamin D metabolites, bisphosphonates, teriparatide, or estrogen, or if they refused to sign an informed consent form. All subjects signed informed consent forms before joining the study. Ultimately 65 patients who underwent KT were enrolled in this study for further analysis. The Protection of Human Subjects Institutional Review Board of Tzu-Chi University and the Hospital approved this study, and all study procedures were conducted in accordance with the principles stated in the Declaration of Helsinki (IRB108-96-B).

### 2.2. Anthropometric Measurements

Trained staff checked and recorded all subjects’ anthropometric parameters, including body weight, body height, waist circumference, body mass index, medical history, medication usage, and lifestyles [15,16,17].

### 2.3. Brachial–Ankle Pulse Wave Velocity and Blood Pressure Measurement

Systolic blood pressure (SBP) and diastolic blood pressure (DBP) were measured by an automatic upper-arm oscillometer device operated by trained staff after subjects rested for at least 10 min. Blood pressure was checked three times via the right brachial artery. A subject was diagnosed to have hypertension if SBP was ≥140 mmHg and DBP was ≥90 mmHg or if the person took antihypertensive medication in the past two weeks. Diabetes mellitus (DM) was defined as fasting plasma glucose ≥126 mg/dL or if the person was using oral or injected diabetic medication.

The baPWV was assessed by an automated vascular device (VaSera VS-1000; Fukuda Denshi, Tokyo, Japan) [15,16,17], which measured SBP and DBP through the sphygmomanometer cuffs in both upper arms and ankles via an automatic oscillometer method. The subject laid down for five minutes, followed by checking his/her blood pressure, as well as electrocardiographic and cardiac phonographic monitoring. The analyzer automatically calculated the baPWV by measuring the length of an arterial segment between the brachium and ankle divided by the time interval between the brachial and the ankle waveform. According to a previous study, PAS was defined as left or right baPWV >14.0 m/s when the study revealed the significant cut-off value of baPWV values [18].

### 2.4. Biochemical Determination

After an 8–10-h overnight fast, blood samples of about 5 mL were obtained from the 65 patients who underwent KT. The blood samples were centrifuged at 3000× *g* for 10 min for further examination. All the chemical analyses of the blood samples except serum intact parathyroid hormone (iPTH) were performed by an autoanalyzer (COBAS Integra 800, Roche Diagnostics, Basel, Switzerland) and completed within one hour of the collection from the recipients. Data measured included total cholesterol (TCH), triglyceride (TG), high-density lipoprotein cholesterol (HDL-C), low-density lipoprotein cholesterol (LDL-C), fasting glucose, blood urea nitrogen (BUN), creatinine (Cre), total calcium, phosphorus, and serum intact parathyroid hormone (iPTH). The concentration of serum iPTH was measured via enzyme-linked immunosorbent assays (Diagnostic Systems Laboratories, Webster, TX, USA). The estimated glomerular filtration rate (eGFR) was calculated by the modification of diet in renal disease formula.

### 2.5. Liquid Chromatography and Mass Spectrometry Analysis

The serum-free l-carnitine level was determined by high-performance liquid chromatography and mass spectrometry (LC–MS). Firstly, 100 µL of serum sample and 50 mM sodium phosphate dibasic heptahydrate solution were mixed in an Eppendorf pipette (1.5 mL). The solution to be analyzed was extracted by Novum Simplified Liquid Extraction (SLE, Phenomenex, Novum®) and was eluted in ethyl acetate (1.5 mL). The elute was then evaporated to dryness under nitrogen flow, and the residue was reconstituted in 100 µL of methanol for analysis. The study used the Waters e2695 HPLC system, made up of a single quadrupole mass spectrometer (ACQUITY QDa, Waters Corp) and equipped with an analytical column (phenomenex Luna® C18(2) (5 µm, 250 × 4.60 mm, 100Å)). The setting of the temperature of the column was at 40 °C, the flow was 0.8 mL/min, and the injection volume was 30 µL.

The LC–MS analyses were performed using a modified method [19] where the pre-treated samples were subsequently determined in positive (i.e., the l-carnitine) ion mode electrospray ionization (ESI). In this positive ion mode, the mass spectrometer was then operated in full scan at the speed of 50–450 *m*/*z*. The individual masses of each compound (l-carnitine: 162.1 *m*/*z*) were monitored in the single ion recording mode. Data acquisition and processing were performed by the Empower® 3.0 software. The retention time for free l-carnitine was 2.49 min. Quantification of the endogenous compounds was done by measuring peak areas, followed by comparison to a calibration curve of the standard solutions.

### 2.6. Metabolic Syndrome and Its Components

Our study used the definition of metabolic syndrome from the International Diabetes Federation [20]. Participants were regarded as having metabolic syndrome when their waist circumference was ≥90 cm (men) or ≥80 cm (women), and when they met two or more of the following criteria: fasting serum glucose of 110 mg/dL, TG of 150 mg/dL, HDL-C level of 50 mg/dL in women or 40 mg/dL in men, blood pressure of ≥130/85 mmHg or use of antihypertensive medication. Participants were classified as having DM when their fasting plasma glucose level was ≥126 mg/dL, when their two-hour glucose during an oral glucose tolerance test was ≥200 mg/dL, or if they were using hypoglycemic medications.

### 2.7. Statistical Analysis

The Kolmogorov–Smirnov test was used for the determination of the normal distribution of data. Normal distribution of data was expressed as means ± standard deviation (SD) and further comparison of data between patients were done by an independent Student’s *t*-test (two-tailed). Non-normal distributed data were expressed as medians and interquartile ranges, and further comparison of data between patients was done using the Mann–Whitney U test. Analysis by chi-square test was done for data expressed as the number of patients. Multivariable logistic regression analysis was used for examination of independence of variables that were significantly associated with PAS in patients who underwent KT. SPSS for Windows (version 19.0; SPSS Inc., Chicago, IL, USA) was used for data analysis. A *p*-value of less than 0.05 was considered statistically significant.

## 3. Results

Thirty-six patients who underwent KT (55.4%) had PAS, and they were typically older individuals (*p* = 0.041), with longer KT duration (*p* = 0.025), higher waist circumference (*p* = 0.010), SBP (*p* = 0.002), serum TG (*p* = 0.040), insulin level (*p* = 0.002), homeostasis model assessment of insulin resistance (HOMA-IR, *p* = 0.002), while they had lower serum HDL-C (*p* = 0.036), and l-carnitine levels (*p* < 0.001) and a higher percentage of diabetes (*p* = 0.001), hypertension (*p* = 0.033), and metabolic syndrome (*p* = 0.044), when compared with the control group (Table 1 and Table 2).

After adjusting for factors that were significantly associated with PAS in these patients by multivariable logistic regression analysis, we found that the KT duration (odds ratio (OR): 1.028, 95% confidence interval (CI): 1.003–1.054, *p* = 0.029) and serum-free l-carnitine levels (OR: 0.916, 95% CI: 0.842–0.998, *p* = 0.044) were independently associated with PAS in patients who underwent KT (Table 3).

**Figure 1:**
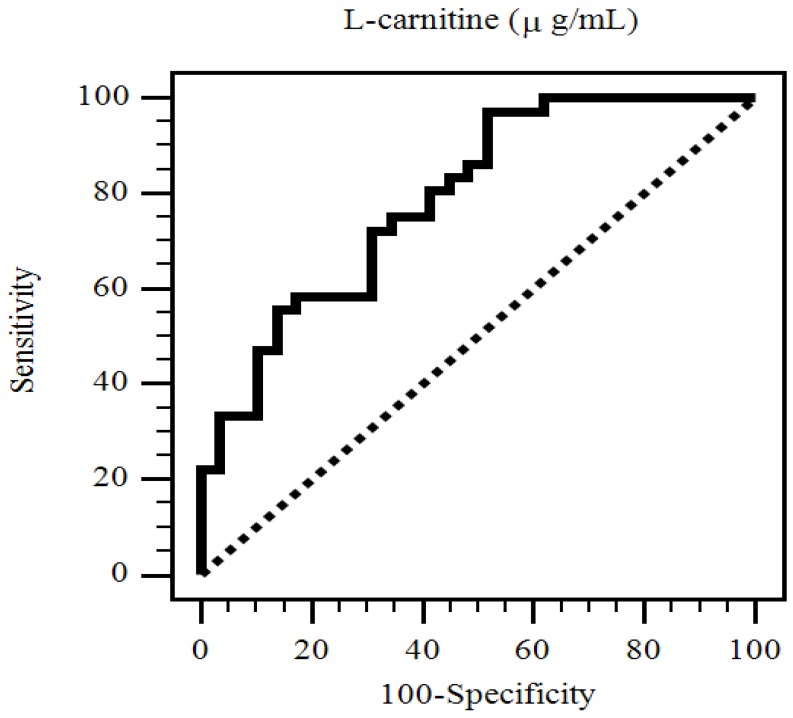
Receiver operating characteristic (ROC) curve analysis to predict peripheral arterial stiffness in 65 renal transplant recipients. The area under the ROC curve (AUC) indicates the diagnostic power of l-carnitine at predicting peripheral arterial stiffness in renal transplant recipients. The AUC for l-carnitine was 0.789 (95% confidence interval: 0.670–0.881, *p* < 0.001).

The area under the receiver operating characteristic (AUC) curve showed that the diagnostic power of l-carnitine in predicting PAS among patients who underwent KT was 0.789 (95% CI: 0.670–0.881, *p* < 0.001) (Figure 1).

## 4. Discussion

The primary novel finding of this investigation was that patients who underwent KT and had high PAS had a concomitant higher prevalence of DM, hypertension, and metabolic syndrome, as well as older age, longer KT duration, higher waist circumference, serum TG, insulin level, and HOMA-IR, while they also had lower serum HDL-C and free l-carnitine levels. After multivariable adjustment, serum-free l-carnitine level and KT duration were independently associated with PAS in patients who underwent KT.

Metabolic syndrome and its components were associated with baPWV values in 2351 Caucasian subjects [21]. Hyperglycemia and high serum insulin levels caused the arteries to become more rigid regardless of the subject’s race or gender in insulin-dependent diabetic patients [22]. Arterial stiffness was positively correlated with serum insulin levels and visceral fat content observed by computed tomography in a study including 2488 older adults [23]. Our results are consistent with previous findings in which patients who underwent KT and had PAS exhibited a higher prevalence of metabolic syndrome and its components, higher serum insulin levels, and HOMA-IR.

Arterial aging is accompanied by vascular remodeling with vascular elastin degeneration, collagen deposition in the arterial wall, endothelial dysfunction, and increases in arterial stiffness [11,24]. During long-term follow-up in patients who underwent KT, progressive arterial stiffening was also found [25]. PAS assessed by baPWV value, most used in East Asian countries, was also confirmed to be associated with cardiovascular risks [26]. Our results also showed a positive correlation between age or KT duration and PAS in patients who underwent KT.

l-carnitine is needed for transportation of long-chain fatty acids into the inner mitochondrial membrane for β-oxidation during fatty-acid metabolism, and it is esterified to form acylcarnitines (ACs) [27]. l-carnitine deficiency induces incomplete fatty-acid oxidation for energy production and impacts gluconeogenesis and increases serum AC levels [27]. Elevated plasma AC levels could result in the alternation of insulin signaling and lead to insulin resistance [28]. l-carnitine can stabilize free radicals via conjugation and prevents the toxic effect of reactive oxygen and nitrogen species, thereby presenting an antioxidant effect [29]. Supplementation of l-carnitine results in better protein balance and improves insulin resistance, anemia, and chronic inflammation status in hemodialysis patients [30,31,32]. A meta-analysis revealed that l-carnitine is associated with a decrease in all-cause mortality compared to placebo for secondary prevention of cardiovascular disease [33]. Arterial stiffness is associated with oxidant stress [34]. Vascular insulin resistance is one of the causes that promote arterial stiffness in obesity [35]. Serum medium- and long-chain dicarboxyl and hydroxyl AC levels were positively associated with PWV in aged adults [36]. Plasma decanoyl carnitine level was positively correlated with baPWV value in men with newly diagnosed type 2 diabetes [37]. Patients with coronary artery disease were found to have a higher serum concentration of ACs [38]. Arterial stiffness was positively associated with cardiovascular events, graft survival, and overall mortality in patients who underwent KT [39]. All these studies indicated that β-oxidation during fatty-acid metabolism is an essential factor associated with arterial stiffness. In our study, serum-free l-carnitine level was independently associated with PAS after adjusting for other cofounders in patients who underwent KT. Although the clinical effect of administration of l-carnitine in KT recipients remains to be assessed, studies showed that it is related to metabolic and inflammatory processes from the molecular aspect [33]. Most researches still showed the protective role of l-carnitine in vascular diseases, and it is recommended that l-carnitine supplementation could be given to in patients with coronary artery disease with a lower level of l-carnitine [40]. However, the benefit of l-carnitine supplementation was not evaluated in the chronic kidney disease group of KT recipients and may need further study for clarification.

There are several limitations to this investigation. Firstly, this was a cross-sectional study, which could not confirm a causal effect between serum l-carnitine and PAS in patients who underwent KT. The relationship between serum l-carnitine and oxidative stress was also not examined in this study. In addition, chronic oral l-carnitine supplementation may increase the formation of trimethylamine *N*-oxide (TMAO) in omnivores, which increases the risk of atherosclerosis [41]. It is worth designing a cohort, randomized control study to confirm the relationship between serum l-carnitine and PAS or oral l-carnitine supplementation and TMAO in patients who undergo KT in the future.

## 5. Conclusions

l-carnitine is an important enzyme co-factor in energy metabolism, and the present investigation demonstrated that the serum fasting l-carnitine level was negatively associated while KT duration was positively associated with PAS in patients who underwent KT. Moreover, we found that the AUC of l-carnitine could predict PAS in patients who underwent KT. Further investigations are required to clarify this relationship. 

## Figures and Tables

**Table 1:** Clinical variables of the 65 renal transplant recipients with or without peripheral arterial stiffness.

Items	All Participants (*N* = 65)	Control Group (*N* = 29)	PAS Group (*N* = 36)	*p*-Value
Age (years)	51.32 ± 9.26	48.72 ± 9.77	53.42 ± 8.40	0.041 *
KT duration (months)	71.97 ± 44.21	58.38 ± 29.37	82.92 ± 51.06	0.025 *
Height (cm)	162.37 ± 8.35	163.38 ± 9.09	161.56 ± 7.74	0.386
Body weight (kg)	63.06 ± 12.32	60.83 ± 10.17	64.86 ± 13.68	0.192
Body mass index (kg/m^2^)	23.88 ± 4.21	22.80 ± 3.48	24.75 ± 4.59	0.064
Waist circumference (cm)	85.56 ± 11.11	81.67 ± 8.84	88.69 ± 11.86	0.010 *
Systolic blood pressure (mmHg)	139.12 ± 16.80	132.07 ± 13.16	144.81 ± 17.40	0.002 *
Diastolic blood pressure (mmHg)	87.28 ± 10.93	84.59 ± 11.24	89.44 ± 10.32	0.075
Left baPWV (m/s)	13.94 ± 2.51	12.02 ± 1.41	15.48 ± 2.11	<0.001 *
Right baPWV (m/s)	14.13 ± 2.69	12.29 ± 1.69	15.62 ± 2.42	<0.001 *
Albumin (mg/dL)	4.14 ± 0.47	4.08 ± 0.53	4.19 ± 0.43	0.346
Globulin (mg/dL)	2.84 ± 0.62	2.81 ± 0.56	2.85 ± 0.67	0.804
Total cholesterol (mg/dL)	195.92 ± 46.34	187.80 ± 37.64	202.47 ± 51.90	0.207
Triglyceride (mg/dL)	120.00 (79.50–175.00)	99.00 (69.50–149.00)	135.50 (84.75–215.50)	0.040 *
HDL-C (mg/dL)	50.88 ± 15.52	55.34 ± 13.19	47.28 ± 16.47	0.036 *
LDL-C (mg/dL)	107.19 ± 35.13	105.12 ± 33.33	108.86 ± 36.90	0.673
Fasting glucose (mg/dL)	94.00 (85.50–111.00)	92.00 (85.00–98.00)	97.50 (86.50–134.75)	0.129
Blood urea nitrogen (mg/dL)	23.00 (17.00–34.50)	19.00 (15.50–30.50)	24.00 (18.00–36.50)	0.101
Creatinine (mg/dL)	1.60 (1.20–2.10)	1.40 (1.10–2.35)	1.70 (1.50–2.10)	0.159
Glomerular filtration rate (mL/min)	43.62 ± 21.74	46.93 ± 23.27	40.94 ± 20.37	0.273
Total calcium (mg/dL)	9.16 ± 1.06	9.25 ± 0.88	9.10 ± 1.20	0.588
Phosphorus (mg/dL)	3.43 ± 0.85	3.37 ± 0.86	3.48 ± 0.85	0.612
Intact parathyroid hormone (pg/mL)	118.20 (75.20–169.05)	118.58 (81.30–177.20)	116.90 (63.08–167.85)	0.329
Insulin (μIU/mL)	9.96 ± 4.65	8.00 ± 3.49	11.54 ± 4.90	0.002 *
HOMA-IR	2.45 (1.41–3.33)	1.84 (1.25–2.62)	2.91 (1.57–3.77)	0.002 *
l-carnitine (μg/mL)	32.04 (23.18–39.25)	36.47 (28.24–54.06)	24.73 (19.40–34.11)	< 0.001 *

Values for continuous variables are given as means ± standard deviation and tested by Student’s *t*-test; variables not normally distributed are given as a medians and interquartile ranges and tested by Mann–Whitney U test. PAS, peripheral arterial stiffness; KT, kidney transplantation; baPWV, brachial–ankle pulse wave velocity; HDL-C, high-density lipoprotein cholesterol; LDL-C, low-density lipoprotein cholesterol; HOMA-IR, homeostasis model assessment of insulin resistance. * *p* < 0.05 was considered statistically significant.

**Table 2:** Baseline characteristics of the 65 renal transplant recipients with or without peripheral arterial stiffness.

Characteristic		Control Group (%)	PAS Group (%)	*p*-Value
Gender	Male	13 (44.8)	22 (61.1)	0.191
	Female	16 (55.2)	14 (38.9)	
Diabetes	No	25 (86.2)	17 (47.2)	0.001 *
	Yes	4 (13.8)	19 (52.8)	
Hypertension	No	19 (65.5)	14 (38.9)	0.033 *
	Yes	10 (34.5)	22 (61.1)	
Transplantation model	Cadaveric	27 (93.1)	29 (80.6)	0.145
	Living	2 (6.9)	7 (19.4)	
Metabolic syndrome	No	23 (79.3)	20 (55.6)	0.044 *
	Yes	6 (20.7)	16 (44.4)	
Tacrolimus use	No	11 (37.9)	15 (41.7)	0.760
	Yes	18 (62.1)	21 (58.3)	
Mycophenolate mofetil or mycophenolic acid use	No	6 (20.7)	12 (33.3)	0.257
	Yes	23 (79.3)	24 (66.7)	
Steroid use	No	4 (13.8)	7 (19.4)	0.546
	Yes	25 (86.2)	29 (80.6)	
Rapamycin use	No	24 (82.8)	28 (77.8)	0.618
	Yes	5 (17.2)	8 (22.2)	
Cyclosporine use	No	23 (79.3)	28 (77.8)	0.881
	Yes	6 (20.7)	8 (22.2)	
Statin use	No	17 (58.6)	20 (55.6)	0.804
	Yes	12 (41.4)	16 (44.4)	
Fibrate use	No	26 (89.7)	29 (80.6)	0.312
	Yes	3 (10.3)	7 (19.4)	

PAS, peripheral arterial stiffness. Data are expressed as number of patients, and analysis was done using the χ^2^ test. * *p* < 0.05 was considered statistically significant.

**Table 3:** Multivariate logistic regression analysis of the factors correlated to peripheral arterial disease among the 65 renal transplant recipients.

Variables	Odds Ratio	95% Confidence Interval	*p*-Value
L-carnitine, μg/mL	0.916	0.842–0.998	0.044 *
KT duration, month	1.028	1.003–1.054	0.029 *
Diabetes, present	4.563	0.171–121.971	0.365
Hypertension, present	2.909	0.137–61.952	0.494
Metabolic syndrome, present	0.053	0.001–2.298	0.127
Age, year	1.056	0.925–1.206	0.422
Waist circumference, cm	1.110	0.984–1.252	0.088
Systolic blood pressure, mmHg	1.068	0.953–1.197	0.259
Triglyceride, mg/dL	1.010	0.997–1.024	0.126
HDL-C, mg/dL	0.941	0.877–1.010	0.093
Insulin, μIU/mL	1.266	0.763–2.102	0.361
HOMA-IR	0.857	0.165–4.449	0.854

* *p* < 0.05 was considered statistically significant in the multivariate logistic regression analysis (adopted factors: diabetes, hypertension, metabolic syndrome, age, kidney transplantation duration, waist circumference, systolic blood pressure, triglyceride, high-density lipoprotein cholesterol, insulin, homeostasis model assessment of insulin resistance, and l-carnitine). LDL-C, low-density lipoprotein cholesterol; HOMA-IR, homeostasis model assessment of insulin resistance.
